# Efficacy of pegylated Graphene oxide quantum dots as a nanoconjugate sustained release metformin delivery system in *in vitro* insulin resistance model

**DOI:** 10.1371/journal.pone.0307166

**Published:** 2024-08-12

**Authors:** Kunal Sarkar, Arindam Chatterjee, Biswabandhu Bankura, Sarbashri Bank, Nirvika Paul, Srilagna Chatterjee, Anwesha Das, Koushik Dutta, Santanu Chakraborty, Sriparna De, Alaa A. Al-Masud, Gausal Azam Khan, Dipankar Chattopadhyay, Madhusudan Das

**Affiliations:** 1 Department of Zoology, University of Calcutta, Kolkata, India; 2 Multidisciplinary Research Unit, Medical College Kolkata, Kolkata, India; 3 Department of Polymer Science and Technology, University of Calcutta, Kolkata, India; 4 Department of Allied Health Sciences, Brainware University, Kolkata, India; 5 Tissue Biobank Section, Research Department, Natural and Health Science Research Center, Princess Nourah Bint Abdulrahman University, Riyadh, Saudi Arabia; 6 Department of Clinical Nutrition, College of Applied Medical Sciences, King Faisal University, Al ASHA, KSA; BRAC University, BANGLADESH

## Abstract

Metformin, the primary therapy for type 2 diabetes mellitus (T2DM), showed limitations such as varying absorption, rapid system clearance, required large amount, resistance, longstanding side effects. Use of Nano formulations for pharmaceuticals is emerging as a viable technique to reduce negative consequences of drug, while simultaneously attaining precise release and targeted distribution. This study developed a Polyethylene Glycol conjugated Graphene Oxide Quantum dots (GOQD-PEG) nanocomposite for the sustained release of metformin. Herein, we evaluated the effectiveness of metformin-loaded nanoconjugate in *in vitro* insulin resistance model. Results demonstrated drug loaded nanoconjugate successfully restored glucose uptake and reversed insulin resistance in *in vitro* conditions at reduced dosage compared to free metformin.

## Introduction

Type 2 diabetes mellitus (T2DM) is the most prevalent and significant metabolic illness that has emerged as a worldwide pandemic in recent years, posing a substantial healthcare challenge globally [1]. Approximately 382 million individuals worldwide had diabetes in 2013 [2]. The incidence of T2DM is steadily rising, and it is estimated that over 590 million individuals will be diagnosed with this ailment by 2035 [3]. Metformin is mostly prescribed to treat T2DM, especially in obese individuals. Metformin has demonstrated a 30% decrease in diabetes mortality and complications when compared to insulin, glibenclamide, and chlorpropamide [4]. Not only that Metformin has been proven to have several new and beneficial roles in recent years. Research has demonstrated that metformin has a significant impact on several malignancies, cardiovascular disease (CVD), liver disorders, obesity, neurological illnesses, and renal diseases. Using the medicine alone or in combination with other drugs been shown useful in treating many ailments [5]. Despite these beneficial effects and becoming a drug for all reasons it has some pharmacokinetic drawbacks. Metformin is shown to have poor bioavailability and rapid elimination through renal and faecal clearance leading to requiring multiple dosing per day with dosing volume reaching up to 3gm/day in some conditions [6]. Also, gastrointestinal intolerance is a significant issue linked to the therapeutic administration of metformin. Around 10% of patients with type 2 diabetes mellitus cannot undergo metformin therapy because of gastrointestinal intolerance symptoms such as diarrhoea, vomiting, abdominal discomfort, and constipation [7,8]. Advancement of Nanotechnology has paved the way forward in circumventing these problems with contemporary medications, such as low bioavailability and rapid drug release into the bloodstream that leads to undesirable side effects [9,10].

In this study we have developed a Polyethylene Glycol (PEG) conjugated graphene oxide quantum dots (GOQDs) based nano drug delivery platform for metformin. GOQDs have demonstrated great drug loading capacity, excellent physiological stability, biocompatibility, strong photoluminescence, and ease of usage, making them a viable nanocarrier for delivering different types of drugs [11–13]. On the other hand, PEG is one of the most used synthetic polymers used in pharmaceutical industry owing to its greater biocompatibility, water solubility, flexibility and non-charge [14]. PEG is also known to increase plasma half life of the drugs by non-interaction with plasma proteins and molecule masking thus increasing bioavailability [15,16]. We have developed Pegylated graphene oxide quantum dots (GOQD-PEG) nanoconjugate to improve the bioavailability of metformin in the system. The study evaluated efficacy of metformin loaded pegylated graphene oxide quantum dots (GOQD-PEG-Met) in *in vitro* insulin resistance model.

## Materials and methods

### Chemicals and reagents

Pristine graphite powder (92% pure), Bis aminated Poly Ethylene Glycol (PEG-NH_2_), N-hydrosuccinamide (NHS), 1-(3-Dimethylaminopropyl)-3 ethylcarbodiimide hydrochloride (EDC), Palmitic acid were procured from Sigma-Aldrich Inc. Metformin Hydrochloride was purchased from Abcam. Concentrated nitric acid acid (70%, GR grade), phosphoric acid, potassium permanganate (KMnO_4_ purified), hydrogen peroxide solution (30% H_2_O_2_), ammonia solution (NH_4_OH, 25%) and all other chemicals of analytical grade were procured from Merck. For cell culture study HepG2 cells were purchased from NCCS Pune. DMEM, FBS, Trypsin and other reagents for cell culture were procured from Thermo Fisher Scientific.

### Synthesis of graphene oxide (GO)

Graphene oxide (GO) was produced using the following process. 2 grams of pure graphite powder were mixed with 180 ml of sulfuric acid and 20 ml of phosphoric acid. The mixture was agitated using a magnetic stirrer for 30 minutes. Next, 10 gm of KMnO_4_ was slowly introduced into the mixture, and the reaction is allowed to proceed for 4 hours in an ice bath. The reaction mixture was then heat-treated in an oil bath at 90°C for 6 hours. The mixture is then poured cautiously into 300 ml of ice-cold triple distilled water. Subsequently, 3 ml of H_2_O_2_ was added to the mixture and stirred for 30 minutes. The graphene oxide that was synthesized was rinsed many times with triple distilled water to remove excess salts and acid.

### Synthesis of graphene oxide quantum dots (GOQDs)

The produced GO (200 mg) was combined with 200 ml of HNO_3_ and sonicated for 1 hour. The mixture was then heated under reflux at 90°C in an oil bath for 8 hours. The acid was eliminated using dialysis method using 1000 kDa cut off dialysis membrane for 48 hours after which the pH of the GOQD solution became 7. After that the GOQDs were lyophilized and stored for further use.

### Synthesis of graphene oxide quantum dots-polyethylene glycol (GOQD-PEG) nanocomposite

The grafting of PEG-NH_2_ onto GOQD was achieved through carbodiimide reaction conducted in a reaction medium containing EDC and NHS. 100 milligrams of GOQD was diffused in 50 ml of triple distilled water for 2 hours. Next, 344mg of NHS and 1600mg of EDC in phosphate buffer (pH 7.2) were introduced to the GOQD dispersion and agitated for 3 hours at room temperature to activate the carboxylic groups of GOQDs. Subsequently, 50mg of PEG-NH_2_ was added into the mixture and left to stir overnight under dark conditions at room temperature (pH 7.2). The reaction mixture was rinsed with triple distilled water for several times and then dialyzed using a dialysis membrane with a molecular weight cutoff of 3000 Kda for 48 hours to procure GOQD-PEG. After that the obtained GOQD-PEG was lyophilized for further use.

### Synthesis of metformin loaded graphene oxide quantum dots-polyethylene glycol (GOQD-PEG-Met) nanocomposite

**Fig 1:**
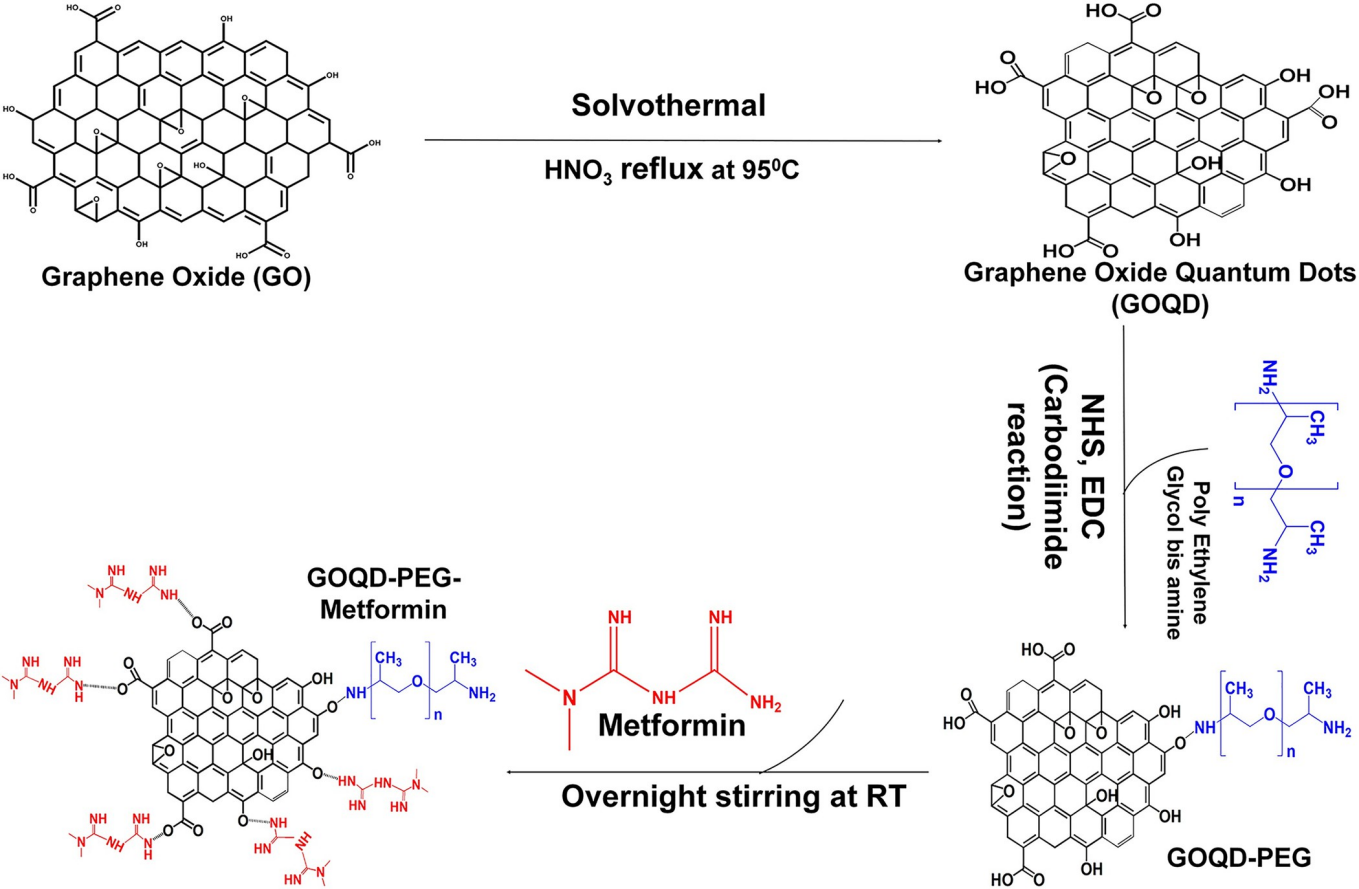
Synthesis scheme of metformin loaded PEGylated graphene oxide quantum dots nanoconjugate.

Metformin was loaded on to the GOQD-PEG by stirring GOQD-PEG (50 mg) with Metformin Hydrochloride (50mg) overnight. The mixture was centrifuged at 10000 rpm for 10 minutes, and the supernatant was collected to calculate the unbound Metformin (Fig 1). The precipitate was collected and freeze-dried for future use. The supernatant collected after the centrifugation step was subjected to UV-Vis spectrophotometry at 234nm for the determination of concentration of unbound metformin. The drug loading was calculated using the formula:

$$Drug\;Loading\;\%=\frac{Total\;amount\;of\;drug-amount\;of\;drug\;in\;supernatant}{Total\;amount\;of\;drug}*100$$

## Drug release

The drug release pattern of metformin from GOQD-PEG-Met was assessed using the dialysis technique at pH 7.4 and 5.4. In brief 50mg of free metformin and GOQD-PEG-Met were dispersed in 25ml of TDW and loaded inside separate dialysis membrane (mol. wt. cutoff 2000 kDa) and the membrane was submerged in the buffer having pH 7.4 and 5.4. 2ml of the buffer solution was removed and replaced with an equal quantity of fresh buffer every hour for 12 hours, and then again at the 24th hour. The buffer solution that was pipetted out was examined using a UV-Vis spectrophotometer to quantify the released metformin at 234nm.

### Characterization

The synthesized GOQD, GOQD-PEG and GOQD-PEG-Met were characterized using UV-vis spectrophotometry, FTIR, TEM, DLS and Zeta potential.

### Cell culture

The HepG2 cell line was obtained from NCCS in Pune, India. The cells were grown in DMEM mix with 10% FBS and 1% penicillin-streptomycin at 37°C with 5% CO_2_.

### Cytotoxicity evaluation of GOQD, GOQD-PEG and GOQD-PEG-Met

MTT assay was performed to evaluate the cytotoxicity of Metformin, GQD-HA and GQD-HA-Met according to [17,18]. In brief, HepG2 cells were seeded in 96 well plates with a 5×10^4^ number and incubated in different concentrations (50μg/ml to 800μg/ml) of GOQD-PEG and GOQD-PEG-Met for 24 h. Subsequently, cells were rinsed three times with PBS. MTT dye (5 mg/ml) was then added to each well, and the plate was incubated at 37°C for 4 hours. Following this, the supernatants were removed, and 200 μl of DMSO was added to each well and gently agitated for 10 minutes to dissolve the formazan crystals. The absorbance was measured at 570 nm with the Varioskan LUX Multimode Microplate Reader from Thermo Fisher Scientific.

### Drug uptake study

HepG2 cells were plated in poly-L-lysine coated coverslips and allowed to grow for 24hr. then 50 μg/ml GOQD-PEG-Met was administered in the culture media. After 8hr the cells were washed with PBS thrice and fixed in 10% paraformaldehyde for 5min, washed with PBS and the coverslips were mounted on a slide. The fixed slides were observed under confocal microscope for cellular uptake of the nanoparticle at 488nm excitation wavelength.

### Insulin resistance model development

Stock solutions of palmitic acid (PA) were prepared by conjugating PA with fatty acid free bovine serum albumin (BSA) [19]. PA was dissolved in preheated 0.1 N NaOH and then diluted 1:10 in prewarmed (45–50°C) DMEM with 12% BSA, resulting in a final concentration of 2.0 mM. The Stock PA solutions were sterilized by filtration and then cells were incubated with 0.25 mM BSA conjugated PA for 24 hours in to induce insulin resistance. Successful induction of IR was confirmed by glucose uptake assay according to [20]. In brief after incubation with PA for 24 hours the cells were washed thrice with PBS and then was cultured in glucose free starvation media for 4 hours. After than starvation media was replaced with DMEM with 1mM glucose and further incubated for 2 hours to asses glucose uptake. After 2 hours of incubation the media was taken and concentration of glucose left out in the media was measured by Glucose oxidase peroxidase method following protocol provided by commercial kit (Arkray Inc.) to assess the glucose uptake percentage in HepG2 cells.

### Dose selection by glucose uptake assay

HepG2 cells were plated in 35mm tissue culture dishes and divided into following groups: control, Insulin resistant (IR), Metformin (0.25mM), Metformin (0.5 mM), Metformin (1 mM), GOQD-PEG-Met (60 μg/ml), GOQD-PEG-Met (120 μg/ml), GOQD-PEG-Met (240 μg/ml). all the groups except the control group was treated with 0.25 mM PA for 24 hours for the induction of insulin resistance. After 24 hours the cells were treated according to the abovementioned treatment dosages and incubated for another 24 hours. Next, the supernatant was collected for estimation of glucose by glucose oxidase peroxidase method by using commercial kit (Arkray Inc.).

### Lipid accumulation assessment by BODIPY staining

Cells were divided into four groups: Control, IR, Metformin (1mM) and GOQD-PEG-Met (120 μg/ml) on coverslips. Then cells were treated with 0.25 mM PA except the control group for 24 hours. After that drug incubation was done for another 24 hours followed by staining with BODIPY. Then cells were observed under fluorescence microscope (EVOS FLoid Imaging Station) for the evaluation of lipid accumulation. The fluorescence intensity was measured by ImageJ software according to [21].

### Gene expression

For gene expression studies we divided the cells into following groups: Control, IR, Metformin (1mM) and GOQD-PEG-Met (120 μg/ml). Then cells were treated with 0.25 mM PA except the control group for 24 hours. After that drug incubation was done for another 24 hours. Following that RNA was extracted from cell lysates using TRIzol reagent from Ambion, life technologies, following the manufacturer’s instructions. 1 μg of total RNA from each sample was reverse transcribed using an iScript cDNA synthesis kit. Gene expression levels in cell lysates were analyzed using quantitative real-time PCR on the Applied Biosystems QuantStudioTM 5 Real-Time PCR System. The forward and reverse primer information for the chosen genes may be found in Table 1. The mRNA expression of the gene was measured using the comparative 2−ΔΔCt technique with GAPDH as the reference gene, following the protocol of Chatterjee et al. [22].

**Table 1:** List of primers.

Gene	Forward	Reverse
**Pepck**	TGGATGTCGGAAGAGGACTTTG	ATACATGGTGCGGCCTTTCA
**G6pc**	ACACCGACTACTACAGCAACA	GACTTCCTGGTCCGGTCTC
**Srebp1c**	AAGCGCTACCGGTCTTCTAT	TTTATTCAGCTTTGCTTCAGTGC
**Hk4**	CATTTCCACCTCACCAAGGA	AAGGAGGGCAGCATCTTAAC
**Pfk1**	GGATGACAAGAGGTTTGACGAG	ATGGCCAGGGAGAAGTTAGA
**Pk**	GACATCGTCTTTGCCTCCTT	GATCTTGATGCCGTGTCCTT
**Gapdh**	CCCTTCATTGACCTCAACTACA	ATGACAAGCTTCCCGTTCTC

### Statistical analysis

The data were analyzed using GraphPad Prism software version 9.3.1 (San Diego, CA, USA), and the findings were reported as Mean ± SEM. One-way ANOVA with Tukey’s post hoc test was utilized to assess statistical significance at different levels of significance (*p < 0.05, **p < 0.01, and ***p < 0.001).

## Results and discussion

**Fig 2:**
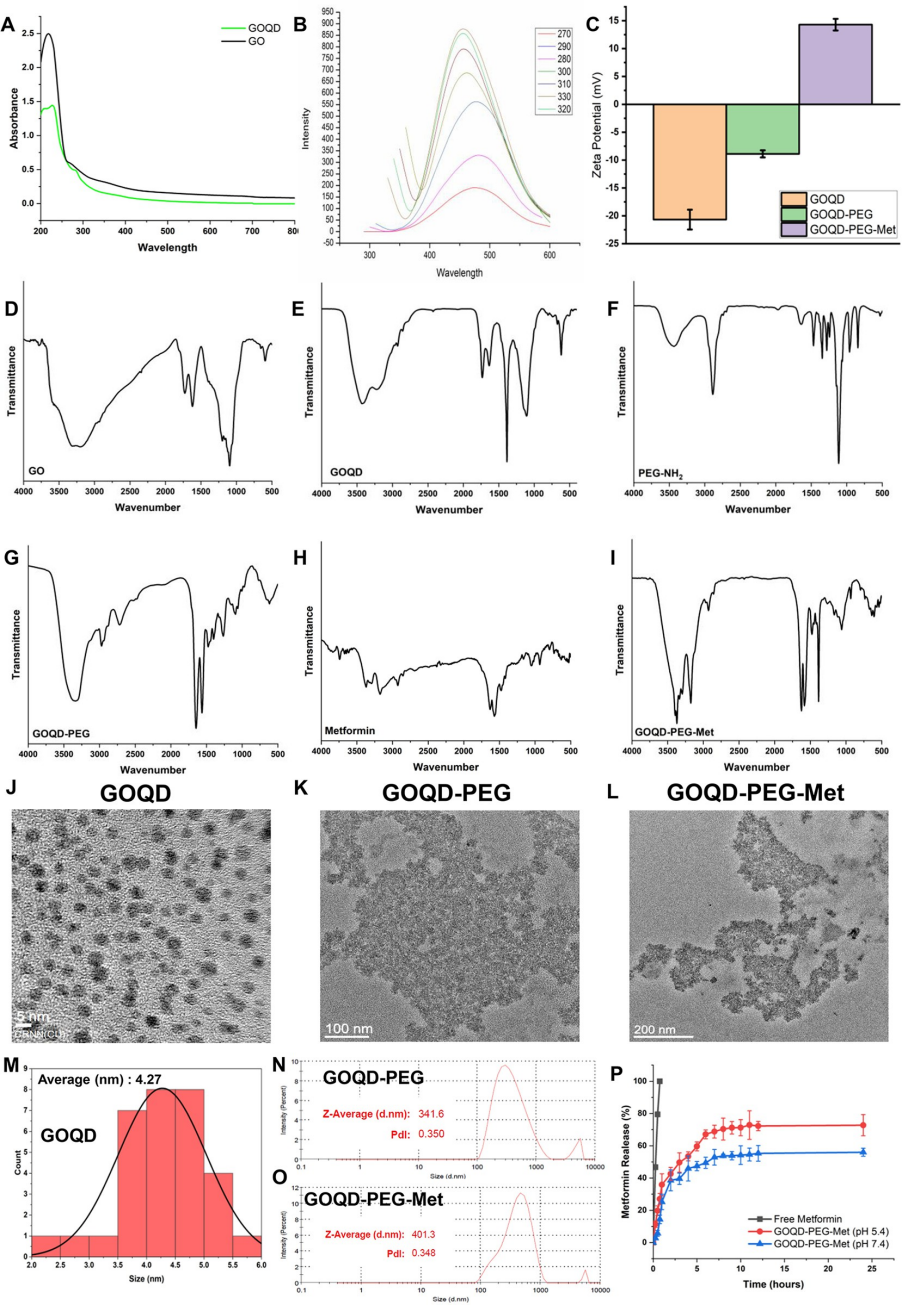
A. UV-Vis spectra of GO and GOQD; B. Photoluminescence spectra of GOQD; C. Zeta potential of GOQD, GOQD-PEG, GOQD-PEG-Met; D.–I. FTIR spectra of GOQD, Polyethylene glycol-NH_2_, GOQD-PEG, Metformin, GOQD-PEG-Met; J.-L. TEM images of GOQD, GOQD-PEG, GOQD-PEG-Met; M. Size distribution of GOQD; N.- O. Average size distribution and PDI of GOQD-PEG and GOQD-PEG-Met; P. In vitro drug release pattern of free metformin and GOQD-PEG-Met.

The GOQD was synthesized by solvothermal method from graphene oxide and then characterization was done using UV-Vis spectrophotometry (**Fig 2A**) where GO shows characteristic peak at 227 nm for π-π* but no noticeable peak observed at 280 nm for n-π* transition. Wheras, GOQD shows peaks for both π-π* and n-π* transition at 227 nm and 280 nm (**Fig 2A**) respectively signifying successful conversion of GO into GOQD [23]. The photoluminescence spectra of GOQD revealed peak fluorescence intensity at λ_em_ 450 nm for λ_ex_ 330 nm **(Fig 2B)**. Whereas TEM images of GOQD **(Fig 2J)** showed round morphology with size range between 2.39 nm to 5.64 nm with average particle size of 4.27 nm (**Fig 2M**) confirming as reported in earlier studies [24]. Next, zeta potential of GOQD was recorded at -20.7 mV **(Fig 2C)**. FTIR spectroscopy of GO (**Fig 2D**) revealed characteristic peaks at 3420 cm^-1^ for -OH functional group, 1726 cm^-1^ (C = O), 1620 cm^-1^ (C = C) and 1096 cm^-1^ for C-O functional group. Furthermore, presence of these peaks in FTIR spectra the of GOQDs **(Fig 2E)** of functional groups like -OH, C = O, C = C, and C-O at 3426 cm^-1^,1725 cm^-1^, 1632 cm^-1^, and 1100 cm^-1^ respectively confirmed the proper synthesis of the GOQD from GO.

After successful synthesis of the GOQD we then grafted the Polyethylene Glycol bis amine via carbodiimide reaction. The FTIR spectra of GOQD-PEG **(Fig 2G)** shows introduction of characteristic peaks like 2889 cm^-1^ for C-H and 1109 cm^-1^ for C-O-C of PEG in the GOQD-PEG nanoconjugate. Also, the peak of C = O at 1725 cm^-1^ disappeared and the 1625 cm^-1^ peak of CO-NH intensified justifying formation of CO-NH bond formation between GOQD and PEG. TEM images of the nanoconjugate revealed amorphous structure with GOQD embedded in the PEG matrix **(Fig 2K)**. Zeta potential of the nanoconjugate was -8.8 due to PEG grafting with GOQD **(Fig 2C)**. The average hydrodynamic particle size of GOQD-PEG was 341.6 nm as determined by DLS study (**Fig 2N**).

Next Metformin was loaded onto the GOQD-PEG nanocomposite by overnight stirring the drug with GOQD-PEG overnight. The FTIR spectra of the GOQD-PEG-Met shows introduction of characteristic peaks of metformin like 3362 cm^-1^ (N-H asymmetric stretching), 3175 cm^-1^ (N-H symmetric stretching), 1474 cm^-1^ (CH_2_ asymmetric deformation) confirming successful loading of metformin **(Fig 2H)**. TEM morphology of the GOQD-PEG-Met showed amorphous structure similar to the GOQD-PEG without the drug **(Fig 2K)**. The zeta potential turned up to +14.29 mV after loading of metformin on the GOQD-PEG nanocomposite **(Fig 2C)**. The drug loading percentage of metformin in GOQD-PEG nanoconjugate was 94.37%. The mechanism of loading of metformin onto the GOQD-PEG surface may be attributed to hydrogen bond formation as metformin contain 4 hydrogen bond donors and 5 hydrogen bond acceptors. GOQDs have several side groups like carboxyl, epoxy and hydroxyl which can form hydrogen bond with amino and imino groups of metformin. Also, the planar structure of GOQD facilitates greater surface area for drug loading than other nanoparticulate drug delivery systems. This property contributes to a higher quantity of drug loading on the GOQD-PEG surface. We have also evaluated the drug release pattern of metformin loaded nanoconjugate compared to free metformin by dialysis method. We observed that the free metformin was released fully within the first hour whereas, the nanoconjugate showed sustained release pattern with 72.76% (pH 5.4) and 55.9% (pH 7.4) drug release after 24 hours of study period **(Fig 2P)**. Relatively higher drug release at pH 5.4 than pH 7.4 can be attributed to the binding of the metformin with GOQD-PEG via hydrogen bonding. The lower pH changes the ionization that leads to breakages in the H-bond between the drug and nanocomposite that results in higher drug release at lower pH, as reported in the previous studies [25,26].

**Fig 3:**
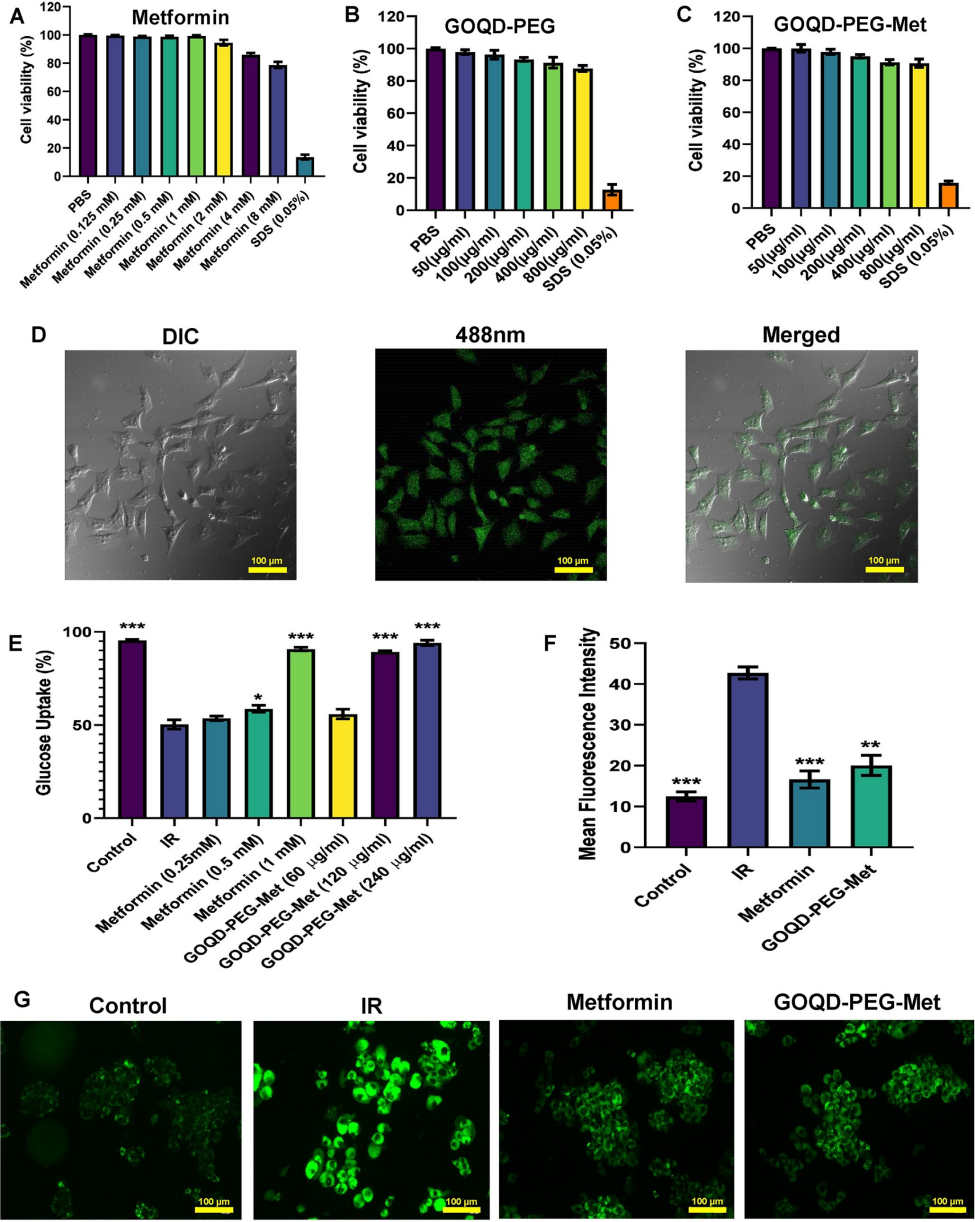
A. B. & C. MTT assay of Metformin, GOQD-PEG and GOQD-PEG-Met in HepG2 cells; D. Confocal laser scanning microscopy images of GOQD-PEG-Met uptake in HepG2 cells; E. Glucose uptake assay and dose selection of GOQD-PEG-Met and Metformin in PA induced *in vitro* insulin resistant model in HepG2 cells; F. BODIPY staining for lipid accumulation study in PA induced HepG2 cells upon GOQD-PEG-Met and Metformin treatment. (n = 3, p values * 0.05, ** 0.01, *** .001, GOQD- Graphene Oxide Quantum Dots, PEG- Polyethylene Glycol, Met- Metformin, IR- insulin resistance).

Overall, toxicity and efficacy studies play a crucial role in optimizing nanoparticle drug delivery systems. Balancing these factors is essential for the successful development of efficient and safe drug delivery platforms. Moving forward, further research in this area will continue to advance the field and improve patient outcomes. We have studied the biocompatibility and efficacy of the synthesized GOQD-PEG-Met in HepG2 cells. HepG2 cells are a well-known in vitro model for drug testing for many years as it mimics hepatic metabolism in vitro [27]. First, we have performed MTT assay to assess cytotoxicity of the nanoconjugate. The MTT assay’s simplicity, cost-effectiveness, rapidity, and quantitative nature make it an attractive option for many researchers. Its compatibility with high-throughput screening and versatility across different cell types further enhances its utility in a variety of research and drug development applications. While acknowledging its limitations such as the need for careful optimization and potential interference by certain compounds., the advantages of the MTT assay ensure its continued relevance in cell viability and cytotoxicity studies [28]. Both the nanoconjugate without the drug (GOQD-PEG) and with drug (GOQD-PEG-Met) showed satisfactory biocompatibility in vitro with 87.72% and 90.56% cell viability respectively in HepG2 cells treated at highest dose of 800 μg/ml **(Fig 3B and 3C)**. Afterwards, cellular uptake of the drug loaded nanocomposite was confirmed by confocal microscopy in HepG2 cells treated with 100 μg/ml GOQD-PEG-Met showing green fluorescence **(Fig 3D)**.

In this study we have used in vitro model of insulin resistance by treating the HepG2 cells with 0.25mM palmitic acid. Palmitic acid is the most abundant free fatty acid present in the body. Elevated amounts of circulating free fatty acids are believed to play a crucial role in triggering and advancing the development of insulin resistance [29]. In our study treatment of the cells with 0.25mM PA for 24 hours caused significant reduction in glucose uptake in the IR group compared to control group. When the PA induced cells were treated with different concentration of free metformin (0.25mM, 0.5mM and 1mM) and GOQD-PEG-Met (60 μg/ml, 120 μg/ml, 240 μg/ml) it was observed that only the 0.5 mM and 1mM free metformin, whereas, 120 μg/ml and 240 μg/ml GOQD-PEG-Met were able to significantly reinstate the glucose uptake **(Fig 3E)**. It is interesting to point out that 120 μg/ml GOQD-PEG-Met which contained equivalent to 0.5 mM metformin restored the glucose uptake more significantly than the 0.5 mM free metformin treated group when compared to PA induced IR group. So, from this observation it can be opined that GOQD-PEG-Met requires lower dose of metformin to exert its efficacy than the free metformin due to delivery of metformin in the nanoconjugate formulation in case of GOQD-PEG-Met. In line with this experimental evidence further studies were carried out using 1mM free metformin and 120 μg/ml GOQD-PEG-Met. We performed BODIPY staining of HepG2 cells for the lipid accumulation study. Here we found that there is a significant increase in the lipid accumulation in the PA treated IR group when compared to control characterized by increased fluorescence **(Fig 3F)**. Whereas, the lipid droplet size and quantity decreased upon treatment of free metformin or GOQD-PEG-Met in the PA treated HepG2 cells. So, apart from improving glucose uptake the GOQD-PEG-Met also efficiently reduced the lipid accumulation in the PA treated cells.

**Fig 4:**
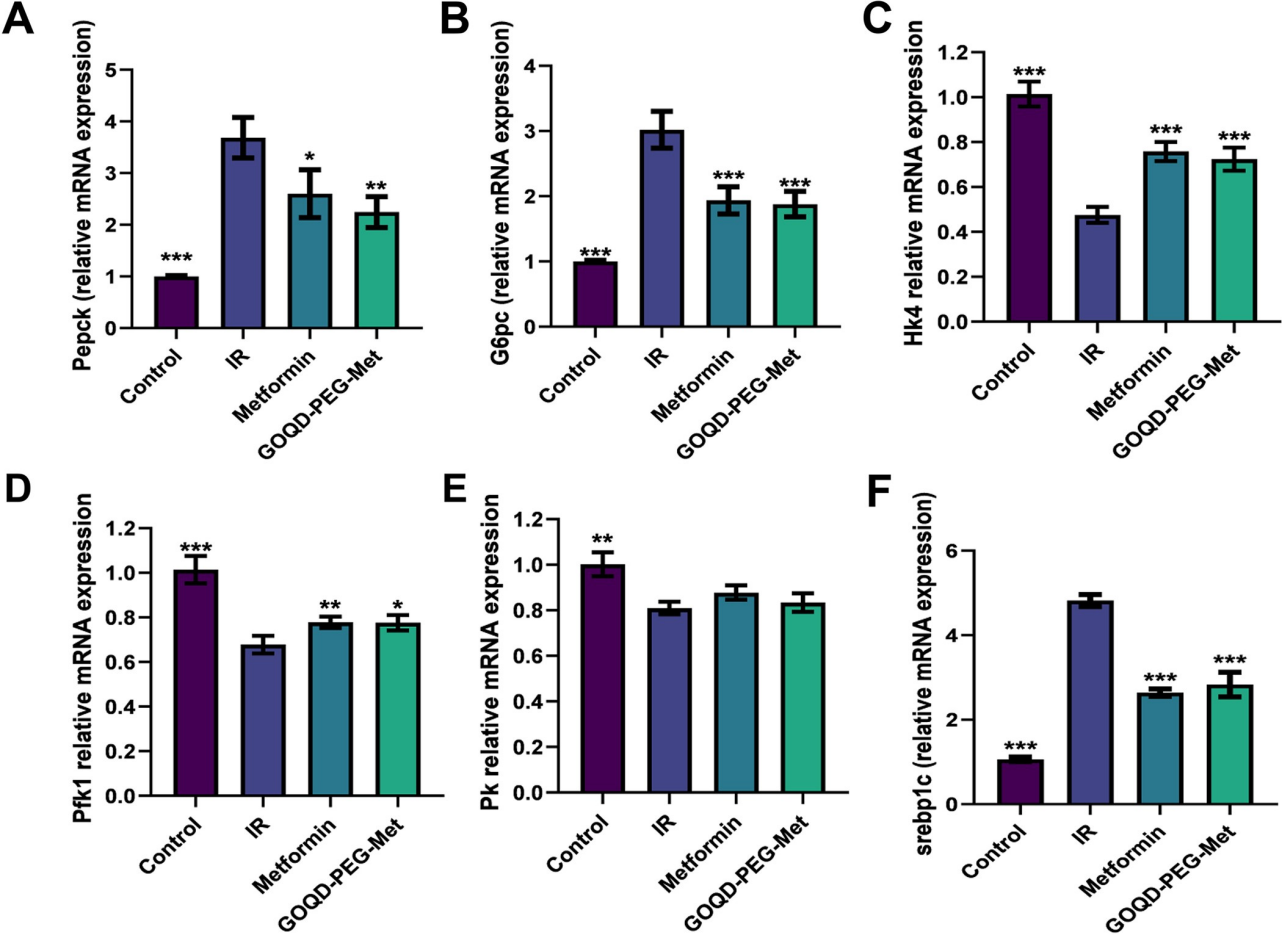
A.-F. Gene expression study of gluconeogenetic, glycolytic and de novo lipid synthesis enzymes in GOQD-PEG-Met and Metformin treated PA induced *in vitro* insulin resistant model in HepG2 cells. ((n = 3–5, p values * 0.05, ** 0.01, *** .001, GOQD- Graphene Oxide Quantum Dots, PEG- Polyethylene Glycol, Met- Metformin, IR- insulin resistance, Pepck- phosphoenol pyruvate carboxy kinase, G6pc- Glucose 6 phosphatase, Hk4- hexokinase 4, Pfk1- phosphor fructo kinase 1, Pk- pyruvate kinase, Srebp1c- sterol regulatory element-binding transcription factor 1).

Furthermore, we studied the gene expression of some key genes related to glucose and lipid metabolism that are regulated by metformin. We observed that mRNA expression of PEPCK and G6Pase the rate limiting enzymes of the gluconeogenesis was upregulated in the IR group. On the contrary the expression of these two genes were significantly downregulated in the GOQD-PEG-Met group when compared to IR group **(Fig 4A & 4B)**. Gluconeogenesis ensues when there is a shortage of glucose in the cells, in this case due to the lowering of glucose uptake as a result of PA induced insulin resistance [30]. The downregulation of the gene expression of key gluconeogenesis pathway gene in the GOQD-PEG-Met group, thus adds weight to the previous finding of the glucose uptake study where glucose uptake was reinstated in the said group of treated cells. As from the aforementioned studies it was confirmed that the GOQD-PEG-Met treatment improved the glucose uptake in the PA treated cells and also downregulated gluconeogenetic pathway genes. Next, we studied whether there were any changes in the glycolytic pathway gene expression because metformin is known to upregulate key glycolytic enzyme activity and transcription [31–33]. So, we checked Hexokinase 4, Phosphofructokinase 1 and Pyruvate kinase mRNA expression in PA treated HepG2 cells compared to control cells and a significant decrease in the gene expression of these genes were observed (Fig 4C, 4D and 4E). Interestingly both metformin and GOQD-PEG-Met treatment significantly reversed the effect of PA on the Hexokinase 4 and Phosphofructokinase 1 mRNA expression. Although the GOQD-PEG-Met was able to achieve similar level of restoration of gene expression of these genes but at lower doses than free metformin. Although pyruvate kinase gene expression was also upregulated in the free metformin and GOQD-PEG-Met group compared to IR group but the changes were not statistically significant. We have also checked the expression of SREBP1c which is involved in the de novo lipid synthesis thus contributes in the lipid accumulation in the cells [34]. Evidently, here we observed a marked increase in the SREBP1c mRNA expression in PA treated IR group compared to control which is significantly downregulated in case of metformin as reported in previous studies [35,36] and GOQD-PEG-Met treated groups **(Fig 4F)**. Findings of the SREBP1c gene expression was in line with the data obtained in the lipid accumulation study where as previously mentioned we observed a stark decrease in both the lipid droplet size and quantity in the metformin and GOQD-PEG-Met treated cells compared to PA treated IR group of cells.

From the abovementioned studies it is imperative that the GOQD-PEG-Met has an edge over free metformin in terms of efficacy and dosing which is very common phenomena in case of nanoparticle based drug delivery system due to enhanced permeability and retention effect. Numerous researches have been undertaken on the delivery of metformin using nanocarriers, both for the treatment of diabetes and cancer [37–40]. Our study distinguishes itself in the field of drug loading and dose reduction by demonstrating that our GOQD-PEG-Met nanocomposite exhibits a substantially greater capacity for drug loading compared to recent studies utilizing nanocarrier-based drug delivery platforms for metformin. This enhanced drug loading capacity is attributed to the utilization of graphene as a nanocarrier, which possesses a planar surface that provides a larger area for drug loading.

## Conclusion

The objective of this work was to design a nanocomposite consisting of graphene oxide quantum dots and polyethylene glycol for the purpose of delivering metformin. The synthesized nanocomposite exhibits a consistent and prolonged drug release pattern in an in vitro drug release assay. We assessed the effectiveness of the GOQD-PEG-Met nanocomposite in a model of insulin resistance induced by PA in HepG2 cells. Our findings indicate that the nanocomposite may achieve the same level of effectiveness as free metformin in enhancing glucose uptake, albeit at two times reduced dosages. Comparable findings were noted in both the investigation on lipid accumulation and the assessment of gene expression. Our investigation has shown that our synthesized nanocomposite is capable of delivering metformin in a sustained release pattern. Furthermore, it has demonstrated greater efficiency compared to free metformin, requiring fewer doses.

At the early phase of development, all studies possess inherent limitations. While we have a high level of confidence in the pharmacodynamics of our PEGylated GOQD-metformin nanocomposite at *in vitro* stage, we do acknowledge a limitation in terms of animal model validation. Cell line research is the preferred and recognized choice at early step of drug development. Pre-clinical experiments may be conducted to evaluate the efficacy of our nanocomposite using an in vitro model. Our synthesized nanocomposite is an innovative approach that has the potential to be administered in insulin resistance condition as a potentially effective therapy for diabetes.

## Supporting information

S1 File Triplicate CLSM images of GOQD-PEG-Met uptake in HepG2 cells. (PDF)

S2 File Triplicate fluorescence microscopy images of BODIPY staining of HepG2 cells for the evaluation of lipid accumulation. (PDF)
