# Supplementary material for: Efficacy of pegylated Graphene oxide quantum dots as a nanoconjugate sustained release metformin delivery system in in vitro insulin resistance model
Source: PLoS One. 2024 Aug 12;19(8):e0307166. doi: 10.1371/journal.pone.0307166 (PMC11318915; doi:10.1371/journal.pone.0307166)
Supplement: S1 File — (PDF) [file pone.0307166.s001.pdf]

Supporting information

Efficacy of Pegylated Graphene Oxide Quantum Dots as a nanoconjugate sustained release Metformin delivery system in *in vitro* insulin resistance model

Kunal Sarkar[1], Arindam Chatterjee[1], Biswabandhu Bankura[3], Sarbashri Bank[1], Nirvika Paul[1], Srilagna Chatterjee[1], Anwesha Das[1], Koushik Dutta[2], Santanu Chakraborty[1], Sriparna De[4], Alaa A. Al-Masud[5], Gausal Azam Khan[6], Dipankar Chattopadhyay[2], Madhusudan Das[1*]

[1]Department of Zoology, University of Calcutta, 35 Ballygunge Circular Road, Kolkata-700019, India. ***Correspondence:** madhuzoo@yahoo.com

[2]Department of Polymer Science and Technology, University of Calcutta, 92 A.P.C. Road, Kolkata- 700009, India.

[3]Multidisciplinary Research Unit, Medical College Kolkata, Kolkata -700073, India

[4]Department of Allied Health Sciences, Brainware University, Kolkata- 700129, India.

[5]Tissue Biobank Section, Research Department, Natural and Health Science Research Center, Princess Nourah bint Abdulrahman University, P.O.Box 84428, Riyadh 11671, Saudi Arabia

[6]Department of Clinical Nutrition, College of Applied Medical Sciences, King Faisal University, Al ASHA, KSA.

Supporting information

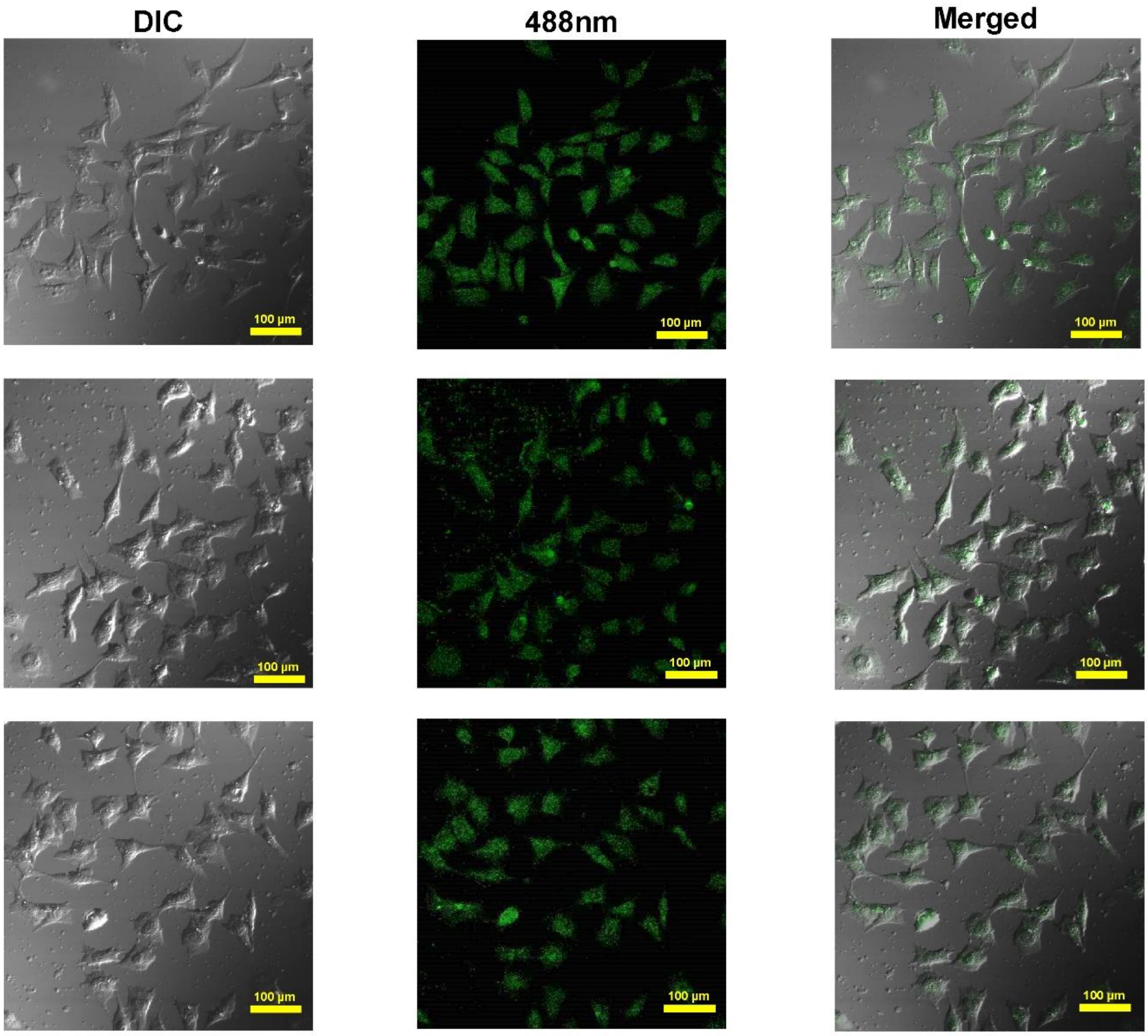

S1 Fig. Triplicate CLSM images of GOQD-PEG-Met uptake in HepG2 cells.
