# Supplementary material for: Efficacy of pegylated Graphene oxide quantum dots as a nanoconjugate sustained release metformin delivery system in in vitro insulin resistance model
Source: PLoS One. 2024 Aug 12;19(8):e0307166. doi: 10.1371/journal.pone.0307166 (PMC11318915; doi:10.1371/journal.pone.0307166)
Supplement: S2 File — (PDF) [file pone.0307166.s002.pdf]

Supporting information

Efficacy of Pegylated Graphene Oxide Quantum Dots as a nanoconjugate sustained release Metformin delivery system in *in vitro* insulin resistance model

Kunal Sarkar[1], Arindam Chatterjee[1], Biswabandhu Bankura[3], Sarbashri Bank[1], Nirvika Paul[1], Srilagna Chatterjee[1], Anwesha Das[1], Koushik Dutta[2], Santanu Chakraborty[1], Sriparna De[4], Alaa A. Al-Masud[5], Gausal Azam Khan[6], Dipankar Chattopadhyay[2], Madhusudan Das[1*]

[1]Department of Zoology, University of Calcutta, 35 Ballygunge Circular Road, Kolkata- 700019, India. ***Correspondence:** madhuzoo@yahoo.com

[2]Department of Polymer Science and Technology, University of Calcutta, 92 A.P.C. Road, Kolkata- 700009, India.

[3]Multidisciplinary Research Unit, Medical College Kolkata, Kolkata -700073, India

[4]Department of Allied Health Sciences, Brainware University, Kolkata- 700129, India.

[5]Tissue Biobank Section, Research Department, Natural and Health Science Research Center, Princess Nourah bint Abdulrahman University, P.O.Box 84428, Riyadh 11671, Saudi Arabia

[6]Department of Clinical Nutrition, College of Applied Medical Sciences, King Faisal University, Al ASHA, KSA.

Supporting information

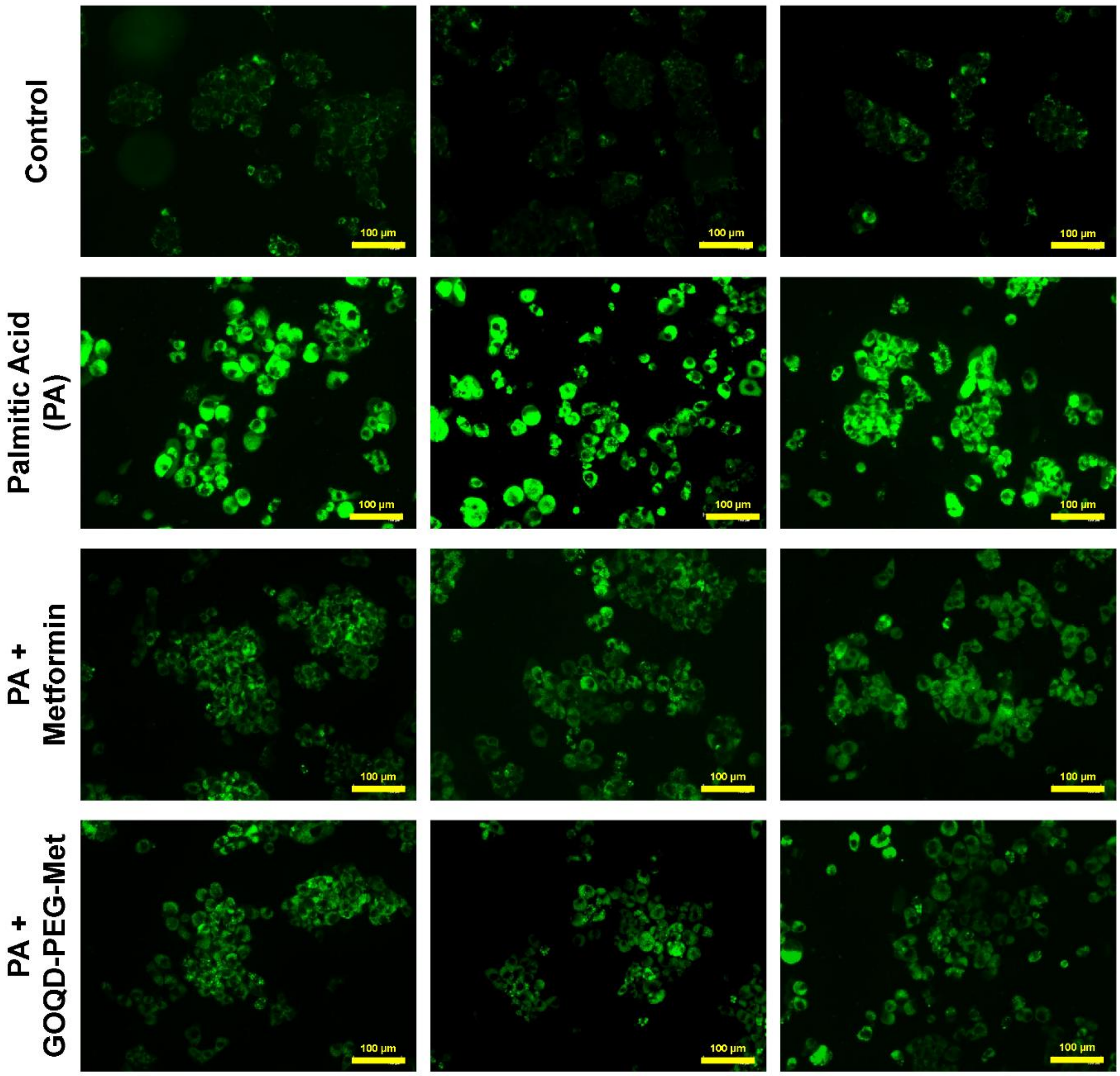

S2 Fig. Triplicate fluorescence microscopy images of BODIPY staining of HepG2 cells for the evaluation of lipid accumulation.
